# Forward-looking activities: incorporating citizens’ visions: A critical analysis of the CIVISTI method

**DOI:** 10.1007/s10202-012-0121-6

**Published:** 2012-11-15

**Authors:** Niklas Gudowsky, Walter Peissl, Mahshid Sotoudeh, Ulrike Bechtold

**Affiliations:** 1 Department of Anthropology, University of Vienna, Althanstraße 14, 1090 Vienna, Austria; 2 Institute of Technology Assessment, Austrian Academy of Sciences, Vienna, Austria

## Abstract

Looking back on the many prophets who tried to predict the future as if it were predetermined, at first sight any forward-looking activity is reminiscent of making predictions with a crystal ball. In contrast to fortune tellers, today’s exercises do not predict, but try to show different paths that an open future could take. A key motivation to undertake forward-looking activities is broadening the information basis for decision-makers to help them actively shape the future in a desired way. Experts, laypeople, or stakeholders may have different sets of values and priorities with regard to pending decisions on any issue related to the future. Therefore, considering and incorporating their views can, in the best case scenario, lead to more robust decisions and strategies. However, transferring this plurality into a form that decision-makers can consider is a challenge in terms of both design and facilitation of participatory processes. In this paper, we will introduce and critically assess a new qualitative method for forward-looking activities, namely CIVISTI (Citizen Visions on Science, Technology and Innovation; www.civisti.org), which was developed during an EU project of the same name. Focussing strongly on participation, with clear roles for citizens and experts, the method combines expert, stakeholder and lay knowledge to elaborate recommendations for decision-making in issues related to today’s and tomorrow’s science, technology and innovation. Consisting of three steps, the process starts with citizens’ visions of a future 30–40 years from now. Experts then translate these visions into practical recommendations which the same citizens then validate and prioritise to produce a final product. The following paper will highlight the added value as well as limits of the CIVISTI method and will illustrate potential for the improvement of future processes.

## Abstract (de)

Hellseherei ist eine alte Kunst. Der Glaube an eine vorherbestimmte Zukunft hat zur Entwicklung der skurrilsten Werkzeuge, mit denen sich diese vorhersehen lassen sollen, geführt. Auf den ersten Blick haben vorausschauende Studien auch immer einen Anschein von Wahrsagerei. Im Gegensatz zum Handlesen versuchen wissenschaftliche Methoden aber lediglich Wege zu zeigen, auf denen sich eine offene Zukunft entwickeln kann. Hinter solchen vorausschauenden Studien steckt oft die Motivation, eine breitere Informationsbasis für EntscheidungträgerInnen zu schaffen, die die Möglichkeit haben, die Zukunft aktiv mitzugestalten. ExpertInnen, StakeholderInnen und Laien können verschiedene Wertvorstellungen und Prioritäten haben, woraus sich unterschiedliche Standpunkte zu verschiedenen Fragestellungen ergeben. Die Einbeziehung einer möglichst breiten Basis solcher Standpunkte kann im besten Fall zu robusteren Entscheidungen und Strategien führen. Dabei stellt die Verarbeitung dieser Diversität in eine für EntscheidungsträgerInnen brauchbare Form eine Herausforderung an das Design und die Durchführung jeden partizipativen Prozesses dar. In diesem Beitrag wird eine neue qualitative Methode für vorausschauende Studien vorgestellt (CIVISTI: Citizen Visions on Science, Technology and Innovation; www.civisti.org), die in einem gleichnamigen EU-Projekt entwickelt wurde und kritisch durchleuchtet wird. Die Methode integriert das Wissen verschiedener Gruppen um Empfehlungen für die Forschungs- und Förderungspolitik zu erarbeiten. Der Schwerpunkt liegt dabei auf Partizipation mit klar getrennten Rollen von BürgerInnen und ExpertInnen. Am Beginn des Prozesses entwickeln BürgerInnen Visionen für eine Zukunft in 30–40 Jahren, aus denen ExpertInnen und StakeholderInnen die Empfehlungen generieren. Diese werden dann an die BürgerInnen zur Bewertung zurückgegeben um ein Endprodukt zu erzeugen. Der folgende Beitrag soll Stärken, Schwächen und Verbesserungspotentiale der Methode aufzeigen.

## Résumé

La voyance, c’est un vieil art. Le fait que l’homme a toujours cru en un avenir prédéterminé et prévisible l’a amené à se servir d’outils les plus bizarres telle que la boule de cristal et d’autres. À première vue, les études visionnaires ont toujours aussi une allure de divination. Contrairement à la chiromancie, les méthodes scientifiques visent simplement à nous démontrer les voies sur lequelles pourra se développer un avenir ouvert. Souvent, de telles études visionnaires ont pour but d’élargir l’éventail d’informations pour permettre aux décideurs de façonner leur avenir tel qu’ils le souhaitent. Souvent experts, parties prenantes et laics ont des représentations du monde et priorités divergentes dont découlent alors des attitudes aussi différentes vis-à-vis des divers problèmes. En impliquant autant de ces attidudes divergentes que possible, on arrivera peut-être à des décisions et stratégies plus solides. L’implication de cette diversité dans le concept et la mise en œuvre de la participation présente un défi pour tout décideur. Cet article présente une nouvelle méthode qualitative pour des études prévisionnelles (CIVISTI: Citizen Visions on Science, Technology and Innovation; www.civisti.org) qui a été mise au point dans un projet UE du même nom, ainsi qu’une analyse critique de cette dernière. Cette méthode se sert des connaissances de différents groupes afin d’élaborer des recommendations pour la politique de recherche et de promotion. L’accent est mis sur la participation avec des rôles clairement séparés de citoyens et d’experts. D’abord, les citoyens déploient aux experts et parties prenantes leurs visions d’un avenir dans les 30 à 40 ans à venir. Ceux-ci en déduisent alors leurs recommandations. Dans la suite, celles-ci sont rendues aux mêmes citoyens pour évaluation et pour la création d’un produit final. L’article présent démontre le pour et le contre de la méthode CIVISTI.

## Introduction

With the aim of producing better decisions and fostering legitimacy (Bobbio 2010), participation tries to raise acceptance and integrate different values into the decision-making process. According to Fiorino (1990), there are at least three different rationales for undertaking public participation: substantive, normative and instrumental. The substantive argument states that considering lay assessment of risks often leads to better decisions than merely relying on experts’ judgement. The normative rationale is based on the notion that the public is best qualified to decide on matters that lie in their own interest. Finally, the instrumental argument reasons that decisions that are taken in consent with laypeople are more likely to be legitimate and accepted. Other authors (i.e. Arnstein 1969; Rowe and Frewer 2005; EIPP 2009) discern different levels of inclusion in public engagement practices: from information through consultation to participation. In extreme forms, participation enables citizens to take decisions themselves. From a science and technology studies perspective, Lengwiler (2008) argues that in most cases participatory approaches are aimed at taking decisions on the science policy level and not at actual research practices. The methods used and their intentions are highly diverse and the decision-makers’ motivations for bringing about participatory exercises may vary as well. Nevertheless, a widespread belief indicates that considering a multiplicity of opinions can lead to socially more robust decisions and *“*[*…*] *brings citizens and institutions closer together”* (Monaghan 2007: 124). Exploring several European citizen conferences, three motives for conducting such exercises on the EU-level have been identified: (a) broadening the basis of information on which decisions are made (in addition to political and scientific arguments), (b) increasing the legitimacy of decisions and (c) shaping a European citizen identity (Boussaguet and Dehousse 2009, Boussaguet 2011).

Abelson et al. (2003, p. 241) summarise that *“collective problem solving discussions are the critical element of deliberation”* allowing for the integration of diverse backgrounds, interests and values to arrive at more informed decisions. This integration of diversity, which Surowiecki (2004) refers to as the *wisdom of crowds* can be cited in favour of all three motives.

In Technology Assessment, participatory methods gained significance as *“the relevance of societal dimensions of science and technology was increasingly acknowledged”* (Joss and Bellucci 2002, p. 5), giving rise to a lively conceptual debate on involving the citizens’ normative evaluations into Technology Assessment (Abels 2007). There is a wide set of tools for participatory Technology Assessment (pTA) and new methods are constantly being developed, all having in common that they involve either laypeople, experts or stakeholders or any combination of these groups in *“*{*…*} *considering and evaluating scientific*-*technical issues beyond their purely scientific, technical and economic aspects*—*as done on classical TA*–*to include wider social, ethical and political aspects”* (Joss and Bellucci 2002, p. 6). The term ‘scientific-technical issues’ can relate to different levels in research and development: a product which has already been introduced into the market can be assessed, as well as ideas of what should be developed. Equally the framework conditions in which research and development takes place can be the subject of the assessment. Research policies are an important aspect of these conditions and are therefore of valid interest for pTA activities. The project CIVISTI,^1^ aimed at developing a method to actively involve laypeople, experts and stakeholders in shaping future science and technology policies in the EU, namely the 8th EU framework programme (Horizon 2020). The method, which is presented and critically assessed in this article, addressed this task by providing tangible input for policy-makers in the form of expert and stakeholder recommendations that are based on citizens’ visions of a desirable future. At the end of this process, the same citizens validated and prioritised these recommendations to produce a final product that stays true to the nature of their visions.

In the wide field of futures studies, there are various definitions for terms like foresight, forecasting or forward-looking activities. In this paper, we use the official EC nomenclature according to which “forward-looking activities” refer to various methods that are *“*[…] *mostly foresight and forecast but also technology assessment and horizon scanning”* (*EC*^2011^). In this context, forward-looking activities are meant to inspire new EU policies by providing fresh insights and identifying major societal challenges (EC 2010) through different inputs. Moreover, the CIVISTI method is a valuable contribution to forward-looking activities, as the participants are consciously dealing with futures when they imagine pleasant (or unpleasant) as well as desirable (or threatening) outcomes.^2^

Throughout this article the term *vision* is employed according to the definition used in the CIVISTI project: *“Vision* [*…*] *is a picture or an imagination of a desirable future. A vision can be based upon hopes and dreams*—*but also upon concerns and fears in relation to problems or imagined threats, which we do not want to become future reality. In CIVISTI, the time span of the vision is 30*–*40* *years from now* (Rask and Damianova 2009: 15)*.”* The method allowed citizens to include their expectations of the future, their thoughts and concerns about it into the visions. As a matter of principle this includes the option for formulating both positive and negative visions. Nevertheless, the majority of the visions as formulated throughout the first application of the method are formulated as positive visions.

After giving a brief overview of participatory foresight, we will give a brief presentation of the CIVISTI method’s aims and background in order to shed light on its main characteristics as compared to other methods. In section two, the CIVISTI method is thoroughly described, and sample results of the process conducted are presented and put into context. The actual assessment of the method concerning limits, added values and potential for improvement (section three) is followed by concluding remarks.

### The participatory turn and its influence on foresight

Various authors describe the possible inaccuracy of expert forecasts in topics such as business, sports or crime and how they might fail to be more accurate than predictions by non-experts (List 2006; Goldstein and Gigerenzer 2009; Andersson et al. 2009; Makridakis and Taleb 2009, a.o.). According to the so-called “experts’ dilemma” (Grunwald 2002), different scientific studies on the same subject can lead to opposing results. Decisions taken only on the basis of expert knowledge also face the question of legitimacy and public acceptance. Among other factors (risk technologies, technology failures, etc.), all these considerations have contributed to an increasing loss of confidence in science’s objectivity and credibility among the public.

In response to the growing pressure on the scientific community to be accountable to society, the “participatory turn” (Jasanoff 2003) took place in science. The inclusion of laypeople demonstrates an appreciation of the value of multiple perspectives, interests and types of knowledge. This is especially important if decision-makers are to be supported in issues, which have implications for a wide variety of actors (cf. Rask et al. 2012). An example of the turn is the development of a set of tools for “democratic technology assessment” that were established in the wake of various controversies concerning new technologies. These tools balance equal participation of laypeople and experts and, if procedural rules are adhered to, they can produce a high level of legitimacy and acceptance (Abels and Bora 2004).

The integration of participation into modelling may be seen as another example of a participatory turn: over the last 20 years, a strong tradition of involving different actors in resource management, land use and community planning can be observed (Sieber 2006), and participatory modelling approaches have helped us to understand the function of resource systems and their sustainable management (Ritzema et al. 2010; Jankowski 2009). These processes often use lay knowledge to find new and assess existing criteria, which leads to better elaborated and thus more accurate models.

In futures studies, the turn started early. From the 1960s on, participation became a key term in democratic theory (Pateman 1970) and, around the same time, some futurists started to step beyond the field’s military origins and began integrating participatory methods into forward-looking activities. Parallel to the development of participatory technology assessment, this movement gained momentum in the 1990s and continues to evolve rapidly today (for a detailed history of participation in futures studies see List 2006).

The concept of visioning has been widely used since the 1980s and 1990s, and even became synonymous with public participation. Planners and communities developed many different methods of varying quality, which all share the goal of creating images of the future that serve as guidelines for future development (Shipley 2002). Many methods apply the concept of “Leitbild” to guide heterogeneous participants on the way towards a certain vision. This is done by consciously intersecting desirable and feasible future developments (Dierkes et al. 1992). Beers et al. (2010) define visions as an image of a desirable future, which makes them a valuable tool for identifying the diverse needs and values of citizens thinking of a variety of futures. The CIVISTI method also allows the integration of undesirable images, which even extends the visions’ capacity for identifying needs. *‘*Needs’ are in this paper understood as *“the most fundamental dimensions of human flourishing and are not good, nor bad, not sustainable or unsustainable* […and] *high quality of life is strongly linked to the fulfilment of individual needs* (Grünberger and Omann 2011, p. 3). With regard to long-term planning linked to the concept of sustainability, it is crucial to integrate *‘multiple legitimate needs’* (Sotoudeh et al. 2011) in the form of involving ‘extended peer communities’, especially when decision stakes and systems’ uncertainty are high (Funtowicz and Ravetz 2001). Failing to consider this plurality of legitimate perspectives in the process of shaping future science, technology and innovation policies may exclude legitimate expectations of laypeople in these areas. Using the citizens’ visions, the CIVISTI method’s core aim is to uncover multiple legitimate needs (wrapped in the visions) and, through their analysis by experts and stakeholders, turn them into an accessible basis for decision-making in the form of recommendations. The CIVISTI method provides a verification phase of the experts’ recommendations by the citizens. This aspect covers the general aim of foresight studies as stated by Warnke and Heimeriks (2008, p. 73), according to whom these studies are set out to support a *“continuous policy learning process”* that is not predetermined but open to foster the development of a system which may cope with future uncertainties. The main instrument, therefore, is agenda setting based on societal needs.

### The principles of the CIVISTI method

The CIVISTI method integrates the foresight approach of creative visioning into a participatory technology assessment setting, leading to a forward-looking activity. This approach is able to specify citizens’ ideas of different aspects of the future and translate them into practical input on research, technology and innovation issues for policy-makers. In the course of the CIVISTI project, the CIVISTI method was designed to enable citizens, stakeholders and experts to actively participate in shaping, for instance, future science and technology policies in the EU. Jacobi et al. (2009b, p. 13) refer to the method as *“long*-*term participatory foresight”*. The external evaluation report (Brandstetter et al. 2011, p. 21) concludes:

“CIVISTI is characterised by a thematically very open approach—the citizens should formulate their visions on future developments concerning technology and society. In this sense CIVISTI does not envisage a thorough explication of a particular topic (e.g., genetic engineering). The main objective is rather to identify new topics for research.”

**Fig. 1:**
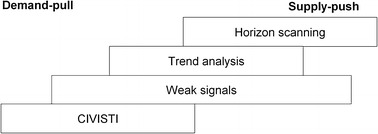
Comparison of the CIVISTI method and other forward-looking methods (adapted from Jacobi et al. 2011)

Applying the CIVISTI method enables citizens to prepare their visions regarding a future 30–40 years from now on the basis of their individual background and their creativity. They integrate hopes and fears as well as norms and values into their contributions. As a first step, participants are asked to think about their own future and the future of their local community, as well as issues related to the future at a wider, e.g., EU or global, level. The only constraints are a given, but very open, format and the editing procedure. The method tries to strip feasibility thinking from the creative process: it takes participants out of their day-to-day life and encourages them to develop their visions as freely as possible. This is not to the same as trying to foresee futures (different possible future options). It should enable citizens to express their views of desirable or undesirable futures. These visions are the starting point for any CIVISTI process. It turned out that the visions are rather problem-oriented descriptions of futures than technological outlooks and the participants most often put societal issues at the centre of their visions. For the processes’ output, it is not relevant whether there is a technology aspect involved in the beginning or not. By handing over the visions to technology and innovation experts in order to create recommendations for S&T policy, the technology aspects take on a more prominent role. Overall, we can say that the CIVISTI method tries to uncover lay perspectives on and expectations of science, technology and innovation by facilitating and mediating between relevant interest groups, such as citizens, experts, stakeholders and policy-makers. Therefore, compared to other forward-looking methods, it is located on the so-called demand-pull side. Other methods that ask for upcoming trends or what technologies could be developed are rather positioned on the supply-push side. Figure 1 illustrates this comparison.

**Fig. 2:**
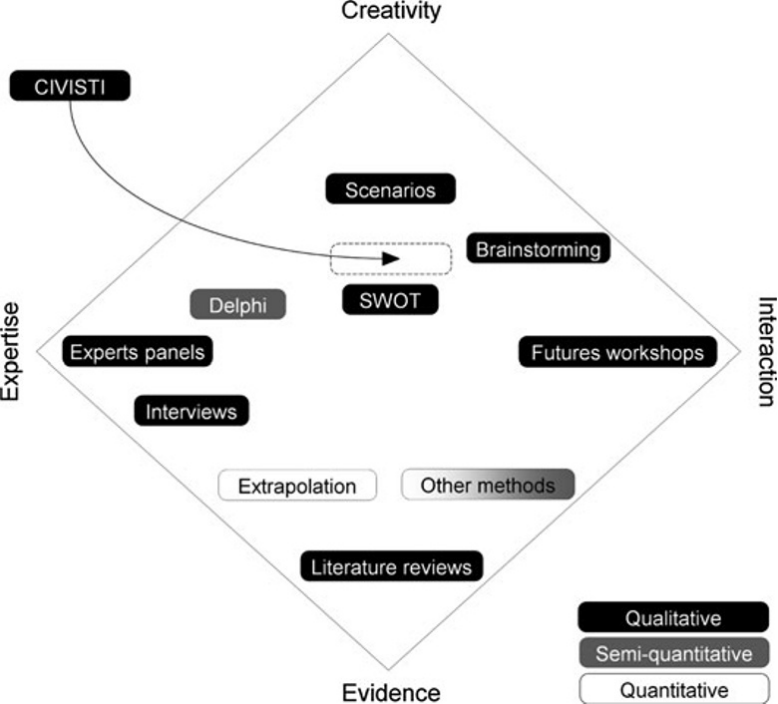
Positioning the CIVISTI method among Europe’s top ten foresight methods (using the *foresight diamond*; adapted from Popper et al. 2007, p. 20)

To further clarify the method’s background and intentions, it is useful to compare CIVISTI with other forward-looking activities. To do so, we use the *“foresight diamond”* adapted from Popper et al. (2007, p. 20) which positions Europe’s top ten methods within a space defined by four opposing poles: evidence and creativity, expertise and interaction. According to this concept, *‘creativity’* is defined by original and imaginative thinking, as applied by individuals or groups of people in, for example, backcasting, essay writing, science fiction literature or brainstorming sessions. Methods that rely heavily on experts’ knowledge would be located close to the ‘*expertise pole’* (i.e., expert panels). ‘*Interaction’* relates to participatory methods that include stakeholders and/or laypeople in foresight activities. An example of a method located close to that pole is futures workshops. Lastly, *‘evidence’*-based methods operate with the support of reliable documentation, statistics or measurement indicators often applied to understand the state of the art of a topic, as in, for example, literary reviews (Popper et al. 2007, p. 20). Figure 2 shows the CIVISTI method’s position in relation to other foresight methods and the four poles. One main focus of the CIVISTI method is the creativity of the participants, since the main component of the process is creative visioning. On the *creativity*-*evidence* axis, we locate the method between *SWOT*^3^ and *Scenarios*, because a second focus of the method is the experts’ contributions, in which, drawing on their knowledge and evidence, the experts moulded the citizens’ work into policy recommendations. This integration of lay and expert knowledge positions the CIVISTI method approximately in the middle of the *expertise*-*interaction* axis.

## Applying the CIVISTI method

The CIVISTI method was developed within an EU project involving seven partner countries, which was coordinated by the Danish Board of Technology. The project was conducted between 2008 and 2011 and was funded under the science and technology foresight call for proposals “Blue Sky Research on Emerging Issues affecting European S&T” in the Seventh Framework Programmes’ (FP7) Socio-Economic Sciences and Humanities theme. The two main aims of the project were (1) to collaboratively develop this new method and test it in the EU context and (2) to use the results to inform the policy-making process of the 8th EU framework programme (Horizon 2020).

In order to provide deeper insight into the method, we will give an overview of the first application of the CIVISTI method during the project of the same name, and then closely examine the genesis of one recommendation throughout the whole process. We will do so by tracking the recommendation that both experts and citizens voted for as their main priority at the end of the project (see Table 1: “attractive public transportation”). Retracing the genesis of a final product backwards to the very beginning of the respective visions it was built upon provides the reader with a practical understanding of both the process and the content.

**Table 1:** Priority lists of citizens and experts on recommendations (according to Jacobi et al. 2011)

Citizens’ voting (all countries)	Experts’ voting
Attractive public transportation	Attractive public transportation
Decentralised energy	Innovations in participation
Re-appropriate the countryside	(European) eco-cities
Tools for disabled people	Recycling complex materials
(European) eco-cities	Ethics of ‘bionic’ production
Social innovation for ageing society	Tools for disabled people
Direct democracy through e-voting	Decentralised energy
Develop effective urban infrastructure	Platform for research in future of work
Policies towards immigrants and refugees	Organic agriculture
Dignity in the dying process	Sofia as an eco-model
Plants for extreme weather	

### From visions to recommendations

**Fig. 3:**
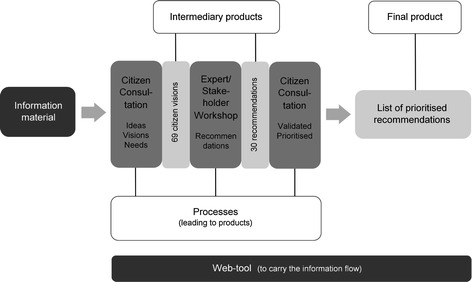
Overview of the CIVISTI method (adapted from Jacobi et al. 2011)

The core of the CIVISTI method is based on three steps: a first citizen consultation, an expert-stakeholder workshop and a second citizen consultation (see Fig. 3). A policy workshop was designed to present and discuss the results with addressees. An external evaluation was performed during all steps of the process.

For the first citizen consultation, a total of 169 citizens from seven EU countries developed their visions, coming together in seven national panels.^4^ Each national panel consisted of about 25 people and met on a weekend during May and June 2009. These groups were not representative for their countries in a statistical sense, but the selection process was adjusted to maximise diversity in terms of demographic criteria such as sex, age, education and occupation. A criterion for exclusion was a professional affiliation to science, technology or politics.

One month before the first citizen consultation, all participants received information on organisational details as well as the CIVISTI magazine^5^*“Eyes on Tomorrow”* in their national language to allow for a common knowledge base. This magazine was designed to provide citizens with diverse expert, stakeholder and lay views upon future developments in Europe to inspire them for their own visions (Brandstetter et al. 2011). During the first citizen consultation, every citizen devised one vision, which was presented to the other participants and voted upon. The top ten visions of each national panel were chosen for further processing. The project team then translated these 69 visions into English and conducted an analysis of the visions’ content, which was based on a grounded theory approach.^6^ This analysis identified 37 topics and served as the basis for developing an analytical model that structured the following expert-stakeholder workshop. The model provided guidelines for extracting the science and technology components and policy options of the identified topics.^7^

In this second step of the process, 18 European technology and innovation experts and stakeholders gathered in June 2010 for a 2-day workshop and transformed the citizens’ visions into 30 recommendations for research agendas and policy options. The participants of this workshop included scientists and policy analysts, non-governmental organizations and representatives of governmental bodies, who were all involved in the making and implementation of research-related policy.^8^ After splitting into groups of three, each team discussed six of the topics that the content analysis had uncovered and which were related to between one and three key visions. They then drafted recommendations (at least one for each vision), resulting in a total of more than 100 recommendations. In an open-space process, experts and stakeholders selected 30 recommendations as the most important, and elaborated them by reflecting on three criteria: novelty, essentiality and timing.

As the third step, the English list of elaborated recommendations was translated into the respective national languages and again returned to the same group of citizens—those who originally devised the visions—in the second citizen consultation. They were asked to validate and prioritise the recommendations according to three criteria:

(1) Faithfulness: to what degree does the recommendation reflect the idea in the vision? Do citizens recognise the vision in this recommendation? Have experts understood the meaning and intention of the vision? (2) Effectiveness: asked whether the recommendation helps to make the vision come true. Could this recommendation be one possible way to support the realisation of the vision? (3) Desirability: To what extent did citizens think the recommendation was desirable or undesirable? (Jacobi et al. 2011, pp. 28–29)

Citizens were then asked to select their seven favourite recommendations. They could not select recommendations that were based on their national visions. Results of the second citizen consultations were translated into English and, finally, the results were presented in a policy workshop at the EU Parliament in January 2011.

The CIVISTI scientific advisory board, the project team, representatives of the EU Commission, and a number of CIVISTI citizens who were interested in discussing the results took part in the policy workshop in Brussels. A poster presentation with results from all partner countries showed highlights of the national processes. After presenting the CIVISTI method and comparing the priorities of the citizens and the experts, participants discussed the results and the future development of the CIVISTI method.

An external evaluation team (Brandstetter et al. 2011) assessed the application of the CIVISTI method during the project using an evaluation method that was based on interviews, document-based research, the participation of evaluators in two national citizen consultations (Austria and Bulgaria) and questionnaires targeting participants of the first and the second citizen consultation. This built-in, yet independent, quality assurance was a valuable resource for both the managers of the process and this paper.

### Exemplary results

#### Prioritising recommendations

At the end of the expert and stakeholder workshop, the participants voted on the recommendations they produced. The citizens also voted on the recommendations in the second citizen consultation. This step was designed to facilitate a comparison of priorities and enable the citizens to give feedback on the experts’ work, so that potential misunderstandings or erroneous interpretations could be detected. For example, a vision on European TV was very popular among the citizens, but they did not prioritise the respective recommendation. They made the criticism that the recommendation had changed the group targeted by the TV channel so that it was predominantly children. Interestingly, there is quite an overlap of recommendations within the top ten. Recommendations that both citizens and experts voted into their top ten are indicated in bold (see Table 1). However, not only are the similarities in how citizens and experts voted important, but the differences between their priorities are also noteworthy (Sotoudeh et al. 2011). For example, in the experts’ votes *‘Innovation in participation’ tied* for first place with the recommendation mentioned above. However, the citizens did not prioritise this recommendation at all. They only partly addressed the issue by prioritising *‘Direct democracy through e*-*voting’*. Taking a closer look at the top ten recommendations of both groups reveals strong democratic values and a strong urge towards sustainability, even though this word did not appear explicitly in the list. Aspects of economic, social, ecological and institutional sustainability are mentioned and thus all four dimensions of sustainability are addressed.

Within the various topics the citizens’ visions addressed, a content analysis identified seven major topics that reappeared frequently: (1) healthcare and medical services; (2) education and learning; (3) ICT, automation and artificial intelligence; (4) legislation; (5) quality of life and life style; (6) employment and innovative modes of work; and (7) energy (Rask and Damianova 2009).

#### Tracing back the results of CIVISTI: the example of “attractive public transportation”

The recommendation “attractive public transportation” is one of the 30 recommendations^9^ that experts developed from the total 69 citizen visions,^10^ which were elaborated during the first citizen consultation. However, to ensure a process unbiased by nationalism, the citizens were not allowed to vote on recommendations that derived from visions which originated in their respective county. “Attractive public transportation” was elected to be of primary importance by experts and citizens. In order to present and examine the process as thoroughly as possible, we will track this recommendation throughout the whole CIVISTI process. Tracing its generic history backwards from the final recommendation to the very beginning of the visions from which it originated shows which parts of the content changed during the process and which remained the same. In the final report and during the policy workshop, which took place in Brussels at the end of the process, the short versions of all recommendations were presented.

**Recommendation: Attractive public transportation (short):**

“Promote technical and social innovations that can enhance people’s access to and use of public transportation: Promote technical and social innovations to improve people’s access to transportation schemes, through an intelligent and interactive network. This network should cover and integrate both local and trans-national travel in a flexible, user friendly and environmentally sound way.”

The elaborated versions of the recommendations were presented to the citizens during the second citizen consultation, before they validated and prioritised them. Besides a description, they also contain the experts’ opinions on novelty, importance and relevancy (timing) of the proposed idea as well as additional comments.

**Recommendation: Attractive public transportation (elaborated)**

“Promote technical and social innovations that can enhance people’s access to and use of public transportation.^11^

*Description of the recommendation*: Promote innovation towards environmentally sound public transportation, by providing individual access to the community-owned and shared transport schemes and vehicles.

*Additional comments from the experts on the recommendation*: Promote technical and social innovations to improve people’s access to transportation schemes, through an intelligent and interactive network. Such a network will promote people’s uses of both traditional public transportation modes and individual access to shared vehicles (e.g., private car sharing). This organic, intelligent, and living (self-adapting) network should cover and integrate both local and trans-national travelling, in a flexible, user-friendly, and environmentally sound way. These innovations should enhance economic productivity by reducing home-to-work travel time, and promote social inclusion, by facilitating the mobility of socially deprived populations.

*Evaluation of the recommendation by the experts*:

• *Novelty*: The idea is quite innovative, since it will involve the use of artificial intelligence to develop a technical and social infrastructure that is focused on the promotion of environmentally sound public transportation.

• *Importance*: Very essential. Solves part of the traffic problems and enhances the current research in environmentally friendly vehicles, because it creates a demand for technology. It solves social problems too for people with poor access, for instance in case of emergency. Faster transportation means increased productivity. This is assumed to promote social inclusion by enhancing mobility for socially deprived people, where lack of transport can be an obstacle to inclusion or access to jobs.

• *Timing*: Very relevant. This should be done immediately. The mental framework is already in place. Scientific and technological solutions exist.”

This recommendation derived from a vision that Danish citizens developed during their national process. The short version presented here provides an overview. The long version^12^ is certainly worth reading to grasp how many different aspects of society a vision on transportation in day-to-day life can relate to.

#### Vision: environmentally sound transportation throughout Europe (short):

“According to our vision, in 2040, all transportation throughout Europe will be environmentally sound, and there will be many environmentally sound means of transportation that can be adapted for the countryside or city, e.g., bicycles, electric cars, electric buses, trams and metro. Public transportation is the most attractive choice and the most popularly used mode of transportation in the individual countries and across borders—even if you are bringing a bicycle, pram or suitcase and need to travel across traffic arteries. Public transportation is fast and easy to access 24 h a day for both shorter and longer distances, and there is no need to use private transportation in cities (Jacobi and Andersen 2009a: 16).”

Differences are observed in the number of issues addressed by the vision and the respective recommendation. The aims also differ: the vision presents a target, whereas the recommendation contains measures required to achieve the kind of public transport system described in the vision.

## Limits, added value and potential for improvement

In this section, we will assess different aspects of both methodological issues and their influence on the results in terms of limits, potential for improvement and added value. Translational problems and the associated loss of information, legitimacy, the citizens’ role and the introductory material as well as methodological novelties will be discussed. At the end of each paragraph possible improvements are identified.

### Methodological novelties

CIVISTI aimed at utilising two distinct resources: (1) the citizens’ visionary ability based upon their individual worldviews, values and experiences that may include hopes, wishes and fears and (2) the experts’ and other stakeholders’ insights into the actual EU R&D-landscape. Building bridges between these two groups and a third one, namely the decision-makers, whose interest in new topics and research programmes allowed for conducting the project in the first place, is the novelty of this method. The process’ structure determined that the experts and stakeholders (almost) never met with the citizens. Their roles were entirely different and the tasks for each group were described transparently during the individual steps of CIVISTI. The iterative cycle of returning the expert-stakeholder recommendations to the group of citizens who created the visions, was integrated to ensure a balanced influence by both laypeople and experts on the results of the process. Also the built-in, yet independent, evaluation is a benefit and provides quality assurance of the process, being a valuable resource for process managers as well as outsiders.

The only limitations for the citizens in terms of visioning were that each vision had to concern a desirable future 30–40 years from now, and it had to be described in a maximum of three pages. Not limited thematically, the visions covered a broad thematic basis:

One of the key characteristics of the visions was the holistic and “interdisciplinary” treatment of future issues. Since expert-based thinking can often be characterized as specialized instead of holistic, and disciplinary instead of interdisciplinary, the visions created by the citizens (or “lay-experts”) in the CIVISTI project are expected to provide new ideas and viewpoints to the experts and stakeholders regarding how to think about and interpret new issues of science and technology policy. (Rask & Damianova, 2009)

This open approach is necessary for eliciting those aspects of citizens’ dreams and concerns with regard to the future that can serve as a base for recommendations (in terms of novelty and timing) and are capable of shaping future research and development. On the other hand, Brandstetter et al. (2011) come to the conclusion that only relatively few visions proposed explicitly new ideas, which had not yet been discussed in societal and political discourses. They state that a more restricted approach (i.e., focussing the topic on innovation in public services) could have led to more radical thinking and possibly to more new ideas. Although the ideas within various visions might not be new—they might even seem irrelevant to the development of a new EU framework programme—sometimes their relation to one another reveals new perspectives. On these grounds, the analysts of the preliminary content analysis identified weak signals, a term coined by Ansoff (1975) and later modified by many others (for an overview see Holopainen and Toivonen 2012). The methodology of identifying weak signals and judging their relevance remains an unsettled issue within futures studies (Miller et al. 2012), but the signals can prove highly relevant to future developments. Herein lays a potential for expanding the CIVISTI process in terms of including a separate, thorough scanning of the visions for weak signals.

### Mediating between lays and experts

Translating laypersons’ visions into experts’ recommendations presented a considerable challenge with regard to the design of the process. In contrast to the citizens, the experts were concerned with feasibility when elaborating the recommendations. One expert stated: *“If something was obviously not feasible experts tried to find solutions which come close to the vision but are feasible”* (Brandstetter et al. 2011, p. 36). This approach can result in a loss of the visionary character original to the citizens’ work. The evaluation report as well as Rask et al. (2010) found that lacking the context of a vision could lead to experts misunderstanding the citizens’ intentions. Jacobi et al. (2009b) state that this delegation of power to experts can harm the authenticity of the results. In some cases (e.g., ‘*European TV*’, described in 2.2), the experts transformed the meaning of the visions’ content and the citizens did not appreciate that, giving low votes for the resulting recommendation. The CIVISTI method integrated this step to allow the citizens to give feedback on the experts’ work. Nevertheless, there is potential for improving the level of understanding between the two groups. In the Austrian process, for example, an expert was invited to the second citizen consultation to report about the process of producing recommendations, so that the procedure of transforming the visions did not remain a black box. Additionally, elected citizens could be invited to the expert/stakeholder workshop to give an overview of their work, thereby eliminating contextual misunderstandings.

In CIVISTI, citizens were expected to develop the future visions and experts were asked to develop recommendations. In the second citizen consultation, citizens pointed out that the recommendations were not as holistic as the visions. One reason for this could be that experts were asked to extract relevant issues from the citizens’ visions and develop recommendations for the EU research programme 2014–2020 without generating their own visions. We could ask whether *“recommendations would be much more holistic if experts were asked to think about a research programme that would start in 2020 and at the same time addresses the visions of citizens for the next 30–40* *years”* (Sotoudeh et al. 2011, p. 20). In such a case, they might be able to approach the visions more openly, or add visionary input themselves. As a result, however, their role would not be completely distinguishable from the role of the citizens.

#### The citizens’ role of ‘lay-experts’

The citizens served the process in the role of “lay-experts”, contributing concerns and expectations regarding an open future and acting as consultants to help prepare the next EU framework programme. They adopted that role with confidence, as 90 per cent of the Austrian participants (70 per cent in Bulgaria) believed that: “research professionals can benefit from the views and experiences of lay-experts and that civic participation ensures that research is guided by common societal values rather than particulate interests (Brandstetter et al. 2011, p. 12).” One-third of the citizens asked for the external evaluation, believed that despite their confidence in the benefits that researchers can gain from engaging with the public, the ability of laypeople to understand complex research topics was limited. As a result, the majority of participants in both countries stated that research and development strategies should be predominantly designed and managed by professional experts. This ambivalence in the citizens’ opinions regarding their knowledge and competence as lay-experts uncovers a potential demand for further supporting their role in such processes (Brandstetter et al. 2011). Here, we see the necessity for better explaining their role to the citizens. They should not be discouraged from participating because they might not understand certain parts of complex research issues. It should be clear that their general interest is sufficient to participate in a process of commonly elaborating demands and challenges for future research, technology and innovation. Another improvement could be the assessment of how policy-makers and experts appraise the laypersons’ ability to make valuable contributions.

There remains the question of why individual citizens, outside of organised civil society, would engage in envisioning a far-off future. There might be numerous reasons, such as curiosity, boredom or simply the promise of a good buffet at the hotel. We assume that a sense of responsibility for the future played an important part. This is reflected in the expectations participants had of taking part in CIVISTI. The majority of citizens stated as their main interest: *“empowering citizens to influence European research issues”* and *“ensuring that research is guided by common societal interests”* (Brandstetter et al. 2011, p. 10). Participatory forward-looking activities should be able to mobilise participants to actively take part in discussions on a future more than 25 years distant.

### Legitimacy and impact

#### Internal legitimacy

Regarding the results of the process with respect to Grunwalds’ (2004) definitions of internal and external legitimacy, we found that internal legitimacy was ascertained through the integrated feedback loop of returning the recommendations for their validation to the same citizens that produced the visions. There were differences between what experts and citizens thought about visions and recommendations: some citizens felt that their vision had not been understood and therefore did not agree with the respective recommendation. Some experts stated that some of the issues addressed in the visions were *“not politically correct”* or *“just stupid”* (Brandstetter et al. 2011, p. 35), but letting both parties vote on the recommendations separately gave them the chance to clearly prioritise which results they valued most and wanted to be the main output of the process. For further CIVISTI exercises, one could discuss the necessity of experts prioritising their recommendations, as the CIVISTI outcome is only determined by the citizens in their second round of validation and prioritisation. Nevertheless, having the opportunity to compare the two top ten lists has proved to be a bonus in terms of providing valuable insight into views on future S&T policy from different perspectives.

#### External legitimacy

To date, the answer to the question of whether the results of the process are accepted by a wider public remains mere speculation. Nevertheless, a diverse group of 169 European citizens, selected according to various socio-demographic criteria (see Sect. 2.1), may raise many of the topics and opinions that a larger, statistically representative group would discuss. With regard to dissemination activities, the results were presented at several academic and non-academic workshops and seminars, at which international and national policy-makers were in attendance. Also several press releases, an EU-Newsletter and a European policy brief^13^ have been released.

Furthermore, non-participating (as well as participating) citizens had a very limited opportunity to follow the path of the CIVISTI results into the decision-making process, because the policy workshop in Brussels took place at the end of the project. Actually only very few citizens attended that workshop, and all of them were already participating in the process. National policy workshops, open to anyone, could improve the communication between citizens and policy-makers and thus improve external legitimacy. Although these events were beyond the scope of the recent CIVISTI process, they could in future provide a useful opportunity for reflection in the CIVISTI process before and after a European policy workshop.

#### Impact

In the Austrian case, some efforts were made to include national policy-makers at an early stage by conducting the second citizen consultation on the premises of the Environment Agency Austria (Umweltbundesamt). Also the chairwoman of the ‘Parliamentary Committee on Research, Technology and Innovation’ was invited to and attended the consultation. This was done for two reasons. Firstly, an early dissemination of results and raising awareness in the governmental body was important, and secondly, it was appropriate to show the citizens that their work was appreciated by political decision-makers.

An assessment of the process’ impact turns out to be weak, concluding that so-called roadblocks on cognitive, structural and operational levels have very little impact on actual policy-making (Rask, forthcoming). This argumentation coincides with Abels’ (2007) account of how many pTA activities fail to have a considerable impact on socio-technological conflicts and their resolution, due to weak links to the political system. Nevertheless, it is very difficult to measure the impact of such a process, even if it were considerably stronger. We cannot be sure to what extent the results had an influence on the decision-makers to whom they were communicated and how this will influence their decisions concerning Horizon 2020. In this respect, one could conclude that conducting such participatory processes has an intrinsic value. Putting participation on the map and keeping it in decision-makers’ as well as citizens’ minds contributes to the development participatory exercises that may, over time, become powerful tools of governance.

### Project management

#### Language issues

The translation of complex visions from seven national languages into English and the translation of English recommendations backwards into national languages must inevitably lead to some losses, because translations were made by the national consortium members, who were research professionals and not translators. Brandstetter et al. (2011) found that some experts, as well as citizens, were negatively surprised by the poor quality of language used in some of the visions and recommendations, respectively. Overall, the language translation was a minor problem, and losses due to this process were kept to a minimum, but this matter could be addressed in future applications of transnational CIVISTI processes by hiring external translators. The trade-off between more accurate translations and the decrease in efficiency of the process through additional costs would have to be explored further. Also, processes conducted at national or regional level avoid this problem, by making translations dispensable.

#### Introduction material

Brandstetter et al. (2011) state that the introductory material for the process was well prepared and provided a common knowledge base for the participants. They also mention that the magazine *“Eyes on Tomorrow”* included diverse views on the future, based upon a variety of valid sources. Its concept was based on the layouts of common popular magazines which is important to maximise the accessibility of informative material. However, according to the evaluation, not all participants read the magazine thoroughly, and the criticism was made that it did not have the appeal of a “practical guide”. It therefore might not have provided the intended orientation for citizens in terms of content (Brandstetter et al. 2011, p. 15).

The information magazine and a short film on major ideas for the future were presented during the introductory phase of the citizen consultation. There are clear advantages to visual messages as they appeal to a wider variety of participants than text does. Nevertheless, it was crucial for the process that the team which assembled the material was international and interdisciplinary, because the same pictures were perceived very differently in different contexts. A picture of palm trees, for example, appealed to the Austrian team very much as a symbol for leisure time and vacation, the Maltese, however, associated this with the busy season, many tourists and a heavy workload.

## Conclusions

From the experiences gained whilst developing and conducting the first CIVISTI process, we conclude the following: CIVISTI is an efficient method for foresight, involving clear and well-balanced features of creativity, interaction and expertise. It results in an improvement in the quality of recommendations for decision-making and is consequently well suited to improve the decisions themselves, by broadening the basis of information on which they are made. Involving citizens and basing the recommendations on their work means that decisions that refer to these recommendations are likely to be more legitimate. This process and allowing citizens from different EU countries work together on issues that indirectly or directly concern themselves may potentially contribute to strengthening EU citizenship. In summary, the CIVISTI process therefore makes a successful contribution with regard to the EU’s reasons for promoting participatory exercises as identified by Boussaguet (2011): (a) broadening the basis of information on which decisions are made (in addition to political and scientific arguments), (b) increasing the legitimacy of decisions and (c) shaping a European citizen identity.

Regarding different future options from the viewpoint of the common notion of political sobriety, as most pointedly expressed by a German politician who remarked “Whoever has visions—needs a doctor”,^14^ leaves a sour taste. But processes like CIVISTI can contribute to strengthen so-called ‘soft methods’, such as visioning and participation by combining them with expert knowledge resulting in tangible policy recommendations. However, CIVISTI is an open process, meaning that we do not know at the start what the citizens’ visions will be and where the experts’ recommendations will lead us. Therefore, conducting such a process is only possible and meaningful in an open political climate, where an arena for transferring such results can be established and addressees appreciate them. It remains unclear to what extent these framework conditions were met.

Independently of these framework conditions that should be met, CIVISTI generates a fairly good pool of results (visions, recommendations and analysis reports) that can be used for further analyses. These results are not only suitable for the intended addressees (EU policy-makers), but may also give rise to interest among the scientific community, technology developers and national, regional as well as local levels of administration (as, indeed, has already happened to some extent). Also, the external evaluation as an integral part of the process was found to be very useful and should be maintained in future applications. In summary, we found that the results gained high internal legitimacy due to the recursive valuation step, which leaves the citizens’ with considerable power over the output of the process.

The method has potential for further development and application to different topics. There are already initiatives for a *‘sustainable city’* approach and another one on health topics in the Austrian context. Another process involving high school students was already conducted in the wake of RIO + 20.^15^ Nevertheless, in terms of the process itself, there is plenty of potential for improvement. Recapitulating the above, we suggest that national policy workshops would be an essential ingredient to foster political interest at an early stage and thus help transfer results to the decision-making level. Furthermore, with regard to the translational loss that occurred, conducting CIVISTI processes on national or lower levels can avoid language-related effects. Concerning the loss of information during the process of transforming citizens’ visions into experts’ recommendations, there is distinct potential for fostering understanding by mixing the two groups to a certain extent, for example, letting citizens address the experts about how their work was structured or contextual issues, as well as inviting experts to the second citizen consultation to help to explain their work. Additionally, an integrated thorough analysis for identifying ‘weak signals’ may further expand the set of results CIVISTI is able to generate. Finally, clearly communicating to the citizens their role as lay-experts may help them to see that their valid interest in and right to express demands towards research, technology and innovation on the basis of their concerns and values are sufficient grounds to take part in such a process.

## Footnotes

1 CIVISTI: Citizen Visions on Science, Technology and Innovation; www.civisti.org, retrieved on 02.04.2012.

2 To conduct this assessment we apply different perspectives to CIVISTI: the outside view, the inside view and the semi-outside view. One author was not involved in the project during its runtime at all. Two of the authors were project managers for the Austrian part of CIVISTI, who contributed with valuable inside information. Finally, a third perspective combines the preceding ones, as one author was only involved as a moderator during the first citizen consultation (Austria), but had no further stake in the project. An additional external view was provided by the evaluation report (Brandstetter et al. 2011).

3 SWOT: an analysis developed to evaluate a company’s strategic position (strengths, weaknesses, opportunities, threats).

4 The partner countries were Austria, Belgium, Bulgaria, Denmark, Finland, Hungary and Malta.

5 The CIVISTI magazine: http://civisti.org/files/images/CVISTI_MAG_ENG1.pdf, retrieved on 02.04.2012.

6 Grounded theory is commonly applied in sociological analysis of qualitative data. It is based on the idea of grounding the analysis as much as possible on the existing data rather than applying predetermined categories. For references see Rask and Damianova 2009, p. 14.

7 For a detailed description of the model see: http://www.civisti.org/files/images/D4_1_Analytical_Model.pdf, retrieved on 29.07.2012.

8 Summary of the expert-stakeholder workshop: http://civisti.org/the_results/expert_stakeholder_workshop, retrieved on 02.04.2012.

9 All recommendations can be found at: http://civisti.org/files/images/Summary_of_the_30_recommendations.pdf, retrieved on 29.07.2012.

10 All visions can be found at: http://civisti.org/the_results/cc1, retrieved on 02.04.2012.

11 This elaborated version of recommendation 18 can be found at: http://civisti.org/files/images/list_of_30_elaborated_recommendations.pdf, retrieved on 02.04.2012.

12 The elaborated version of this vision can be found at: http://www.civisti.org/files/images/Civisti_Danish_Catalogue_in_English.pdf, retrieved on 29.07.2012.

13 EU-Newsletter and a European policy brief: http://civisti.org/publications/other_publications, retrieved 04.04.2012.

14 As stated by Helmut Schmidt in an newspaper interview [translated by the authors]: http://www.welt.de/politik/article2916542/Helmut-Schmidt-berauscht-mit-seiner-Nuechternheit.html, retrieved on 02.04.2012.

15 CIVIISION: http://www.oeaw.ac.at/ita/ebene4/d2-2d42.htm, retrieved on 12.08.2012.
